# The Role of Lipid Competition for Endosymbiont-Mediated Protection against Parasitoid Wasps in *Drosophila*

**DOI:** 10.1128/mBio.01006-16

**Published:** 2016-07-12

**Authors:** Juan C. Paredes, Jeremy K. Herren, Fanny Schüpfer, Bruno Lemaitre

**Affiliations:** a Global Health Institute, School of Life Science, École Polytechnique Fédérale de Lausanne (EPFL), Lausanne, Switzerland; b Molecular Biology and Bioinformatics Unit, Martin Lüscher Emerging Infectious Diseases (ML-EID) Laboratory, International Centre of Insect Physiology and Ecology (icipe), Nairobi, Kenya

## Abstract

Insects commonly harbor facultative bacterial endosymbionts, such as *Wolbachia* and *Spiroplasma* species, that are vertically transmitted from mothers to their offspring. These endosymbiontic bacteria increase their propagation by manipulating host reproduction or by protecting their hosts against natural enemies. While an increasing number of studies have reported endosymbiont-mediated protection, little is known about the mechanisms underlying this protection. Here, we analyze the mechanisms underlying protection from parasitoid wasps in *Drosophila melanogaster* mediated by its facultative endosymbiont *Spiroplasma poulsonii*. Our results indicate that *S. poulsonii* exerts protection against two distantly related wasp species, *Leptopilina boulardi* and *Asobara tabida*. *S. poulsonii*-mediated protection against parasitoid wasps takes place at the pupal stage and is not associated with an increased cellular immune response. In this work, we provide three important observations that support the notion that *S. poulsonii* bacteria and wasp larvae compete for host lipids and that this competition underlies symbiont-mediated protection. First, lipid quantification shows that both *S. poulsonii* and parasitoid wasps deplete *D. melanogaster* hemolymph lipids. Second, the depletion of hemolymphatic lipids using the *Lpp* RNA interference (*Lpp RNAi*) construct reduces wasp success in larvae that are not infected with *S. poulsonii* and blocks *S. poulsonii* growth. Third, we show that the growth of *S. poulsonii* bacteria is not affected by the presence of the wasps, indicating that when *S. poulsonii* is present, larval wasps will develop in a lipid-depleted environment. We propose that competition for host lipids may be relevant to endosymbiont-mediated protection in other systems and could explain the broad spectrum of protection provided.

## IMPORTANCE

Virtually all insects, including crop pests and disease vectors, harbor facultative bacterial endosymbionts. They are vertically transmitted from mothers to their offspring, and some protect their host against pathogens. Here, we studied the mechanism of protection against parasitoid wasps mediated by the *Drosophila melanogaster* endosymbiont *Spiroplasma poulsonii*. Using genetic manipulation of the host, we provide strong evidence supporting the hypothesis that competition for host lipids underlies *S. poulsonii*-mediated protection against parasitoid wasps. We propose that lipid competition-based protection may not be restricted to *Spiroplasma* bacteria but could also apply other endosymbionts, notably *Wolbachia* bacteria, which can suppress human disease-causing viruses in insect hosts.

## INTRODUCTION

Insects commonly harbor bacterial endosymbionts (i.e., bacteria living inside the host) that are transmitted from mothers to their offspring, often in the egg cytoplasm (1). The fitness of these inherited symbionts is intimately tied to that of their hosts, with vertical transmission serving as the key force driving their long-term coevolution. Some endosymbionts are obligate because they are essential for host survival, for instance by supplying their host with essential nutrients that are missing from its diet (2). Other endosymbionts are facultative, since they are not required for host development or survival (1, 3). Despite their imperfect maternal transmission, facultative insect endosymbionts are widespread in insect populations. This contradiction is likely explained by the fact that facultative endosymbionts have developed additional strategies to increase their own transmission, such as manipulating host reproduction (e.g., male killing, parthenogenesis induction, or cytoplasmic incompatibility) or protecting their hosts against natural enemies (4, 5). In insects, symbiont-mediated protection has now been demonstrated against various pathogens, including parasitic wasps, nematodes, RNA viruses, fungi, and *Plasmodium* parasites (6–13). Symbiont-mediated protection is also the cornerstone of a number of symbiont-based control strategies, such as the recent field release of *Aedes* mosquitoes transfected with a strain of *Wolbachia* with the aim to suppress dengue virus transmission (14).

Although the mechanistic bases of protection by endosymbionts remain poorly characterized, three mechanisms have been proposed (5). The first proposes that endosymbionts have the capacity to enhance insect immune responses. This mechanism has been described in the pea aphid (*Acyrthosiphon pisum*) when it harbors a secondary endosymbiont (15, 16). Also, a number of studies have suggested that *Wolbachia* bacteria protect insects against viruses by priming their immune system (17, 18). Nevertheless, this mechanism has been observed only in novel host-endosymbiont associations, in which insect species have been experimentally infected with *Wolbachia* bacteria in the laboratory. In contrast, studies based on natural *Drosophila*-endosymbiont associations have not observed any impact of endosymbionts, including *Spiroplasma* and *Wolbachia* bacterial species, on the host immune system (19–21). The second mechanism involves endosymbionts producing a toxin that targets host enemies. Evidence for the importance of this mechanism is found in the results of several studies. The production of bacteriophage toxins (such as YD repeat toxin) has been proposed to underlie the protection exerted by *Hamiltonella defensa* in aphids against parasitoid wasps (22). Moreover, pederin, which blocks protein and DNA synthesis by endosymbiotic *Pseudomonas* species, has been shown to be involved in the protection of rove beetles against predators (23). Finally, a ribosome-inactivating-protein (RIP) toxin encoded by *Spiroplasma* bacteria has been implicated in the protection of *Drosophila neotestacea* against entomopathogenic nematodes (24). All of these toxins target essential eukaryotic processes, and therefore, it is still unclear what prevents their toxicity to the insect host. The third mechanism is metabolic competition, where the symbiont inhibits the growth of its host’s parasites by depleting resources necessary for their development. The metabolic competition hypothesis is supported by observations that insect endosymbionts and the parasites against which they protect share the same environment: the intracellular compartment for *Wolbachia* and viruses or the hemolymph compartment for *Spiroplasma* and wasp and nematode macroparasites. The metabolic competition theory is very appealing because it does not require a specific interaction between the endosymbiont and the host and, therefore, could explain the broad spectrum of protection provided by endosymbionts. While many authors have discussed the idea of nutrient competition, there is a lack of experimental evidence for it. It is, however, notable that one study has linked *Wolbachia* protection against viruses to a competition for cholesterol (25).

Here, we have investigated the mechanisms by which *Spiroplasma* bacteria protect their host against parasitoid wasps. Our study focuses on *Spiroplasma poulsonii* strain MSRO (*melanogaster* sex ratio organism; isolated from a fruit fly captured in Uganda), a natural endosymbiont of *Drosophila melanogaster* (3, 26). In this work, we provide strong evidence that the protection against parasitoid wasps in *D. melanogaster* provided by *S. poulsonii* is mediated by a competition for host lipids. The genus *Spiroplasma* belongs to a group of wall-less bacterial species called *Mollicutes* that infect a broad range of arthropods (27, 28). *S. poulsonii* resides in large numbers in the hemolymph (the insect “blood”) of larvae and adults. *S. poulsonii* cells are neither detected nor affected by the *D. melanogaster* immune system, but their proliferation is constrained by the availability of hemolymph lipids (19, 29). This dependence on lipids is thought to couple the proliferation of *S. poulsonii* to the nutritional state of its host. To ensure efficient vertical transmission, these bacteria use the yolk uptake machinery to colonize the germ line (30). *Spiroplasma* is also a male killer (the male offspring of infected females die during embryogenesis), and it has been hypothesized that this reproductive manipulation is one of the driving forces that maintains this facultative endosymbiont in fly populations (31). Recently, it has been shown that *Spiroplasma* also confers resistance to parasitoid wasps, which are major macroparasites of *Drosophila* flies (12), although the mechanism underlying this protection has remained unknown.

## RESULTS

### *S. poulsonii* confers protection against two distantly related species of parasitoid wasps.

**FIG 1:**
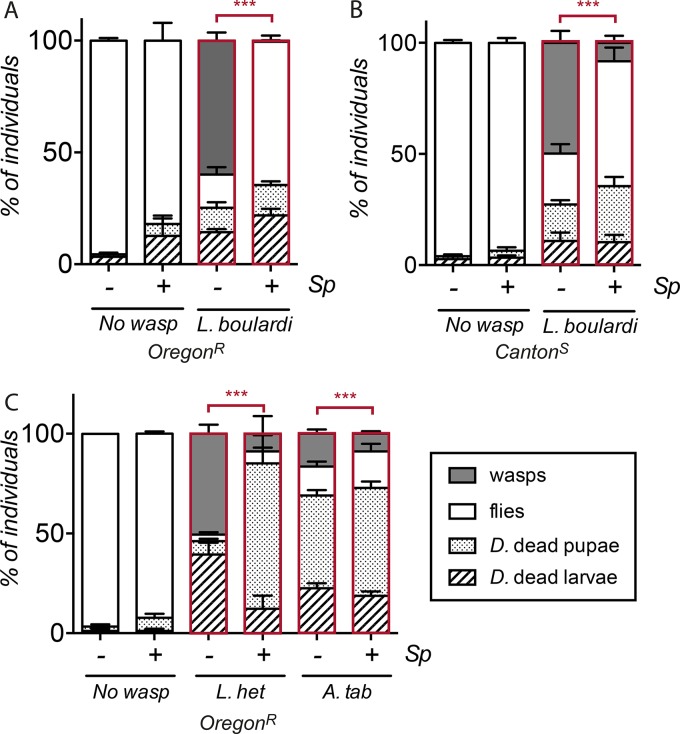
*Spiroplasma poulsonii* confers protection against two distantly related species of parasitoid wasps. Quantification of *D. melanogaster* (*D*.) dead larvae and pupae, emerging fly adults, and wasp adults. (A) *Leptopilina boulardi* infestation in *D. melanogaster* flies with an Oregon-R genetic background (***, *P* < 2.2 × 10^−16^; chi-square = 1,240.5; df = 3). (B) *Leptopilina boulardi* infestation in *D. melanogaster* flies with a Canton-S genetic background (***, *P* < 2.2 × 10^−16^; chi-square = 175.81; df = 3). (C) *Leptopilina heterotoma* (*L. het*) (***, *P* < 2.2 × 10^−16^; chi-square = 180.82; df = 3) and *Asobara tabida* (*A. tab*) (***, *P* < 5.75 × 10^−10^; chi-square = 45.972; df = 3) infections in *D. melanogaster* flies with an Oregon-R genetic background harboring (*+*) or not harboring (*−*) *S. poulsonii* (*Sp*). (A to C) Results are represented as mean percentages ± standard errors of the means (SEM) of a minimum of 270 *D. melanogaster* larvae from three independent experiments. Statistical significance was calculated using Pearson’s chi-square test.

*Leptopilina* wasps (*Figitidae*) are major parasites of *Drosophila*. In the wild, these wasps lays eggs in first- and second-instar (larval stage 2 [L2]) *Drosophila* larvae; the wasp larvae then develop in the host hemolymph during the larval and early pupal stages and hatch from the parasitized pupal case. Previous studies showed that *Spiroplasma* infection enhances the survival of *Drosophila hydei* flies infested with *Leptopilina heterotoma* and of *D. melanogaster* flies infested with *Leptopilina boulardi*. We first extended the studies on the *D. melanogaster*-*Spiroplasma*-*L. boulardi* association (32) by analyzing the impact of *S. poulsonii* strain MSRO in a different host genetic background, *D. melanogaster* strain Oregon-R wild-type flies infested with *L. boulardi*. We monitored the four possible outcomes of infestation: wasp-infested individuals die as larvae or as pupae (both wasp and fly die), a wasp emerges from the pupa (wasp success), or a fly emerges from the infested pupa (fly success). The results presented in Fig. 1A show that more than 60% of infested Oregon-R larvae give rise to an adult wasp, while the other 25% die either as larvae or as pupae. Only 15% of the flies were recovered following *L. boulardi* infestation, consistent with the high virulence attributed to this species (33). Strikingly, the presence of *S. poulsonii* strongly protected *D. melanogaster* against *L. boulardi*, as 60% of the emerging adult insects were flies, and almost no wasps were recovered. The protection exerted by *S. poulsonii* was not caused by a lower infestation rate, as *D. melanogaster*-harboring larvae were infected with an efficiency similar to the infection of larvae devoid of *S. poulsonii* (see Fig. S1 in the supplemental material). The protection phenotype was not linked to a specific genetic background, since we observed the same level of protection in *D. melanogaster* strain Canton-S as in the Oregon-R wild-type strain (Fig. 1B). Interestingly the Canton-S strain used in this study is naturally infected with *Wolbachia*. Thus, the high level of wasp success in Canton-S flies (Fig. 1B) suggests that the intracellular symbiont *Wolbachia* does not confer significant protection against this parasitoid wasp, as previously reported in Xie et al. (32).

*L. boulardi* is a highly specialized parasitoid wasp infecting mostly *D. melanogaster* (34). Thus, the *S. poulsonii*-mediated protection observed could be an outcome of a coevolutionary process and, thus, specific to this species. To analyze whether *Spiroplasma* mediates a broad range of protection against wasps, we performed similar experiments using *L. heterotoma*, a generalist wasp strain from the *Leptopilina* group, and *Asobara tabida*, belonging to the *Braconidae* family, which is phylogenetically distant from the *Figitidae*. The results presented in Fig. 1C show that *S. poulsonii* also confers protection against *L. heterotoma* and *A. tabida*, with a lower percentage of wasps emerging from infested pupae in the presence of *S. poulsonii*. The levels of fly success in the presence of *S. poulsonii* were lower with these two wasp species than was observed with *L. boulardi*; however, the wasp success was strongly reduced*.* This is probably due to the strong pupal lethality (neither flies nor wasps emerge) observed with these generalist wasps, which has also been reported in previous studies (12, 32). Altogether, these findings indicate that *S. poulsonii* can provide broad protection against various wasp species, suggesting a rather general mechanism of protection linked to the biology of parasitoids.

### *S. poulsonii* inhibits wasp growth at the pupal stage.

**FIG 2:**
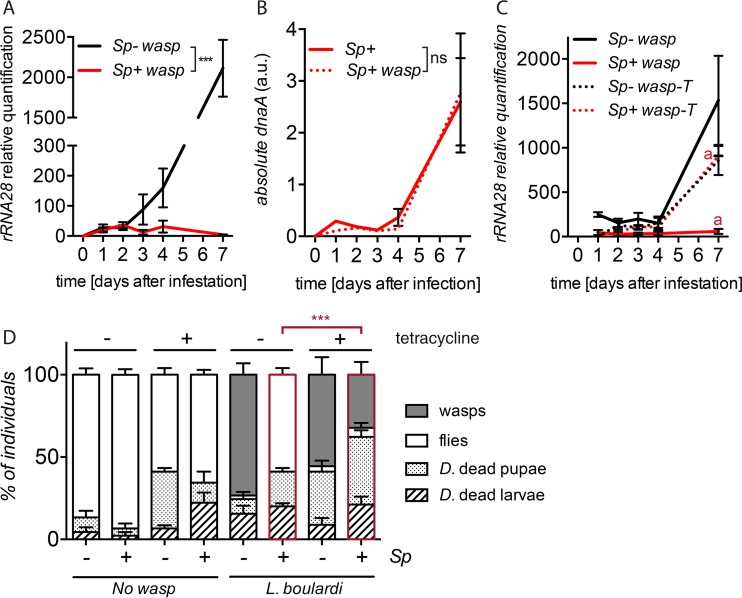
*Spiroplasma* inhibits wasp growth at the pupal stage. (A and C) Quantification of wasp growth was performed by monitoring the amount of wasp 28S rRNA relative to *D. melanogaster RpL32* RNA in *D. melanogaster* larvae/pupae harboring (*Sp+*) or not harboring (*Sp−*) *S. poulsonii*. ***, *P* < 0.001 for comparison of wasp growth in presence or absence of *S. poulsonii* (A); ***, *P* < 0.001; *t* = 24.22; df = 4, for comparison of wasp growth with or without tetracycline treatment at 7 days using unpaired Student *t* test (C). (B) *S. poulsonii* absolute titers monitored by qPCR of the *S. poulsonii dnaA* gene. Wasp infestation has no effect on *S. poulsonii* growth. Not significant [ns], *P* = 0.2867. (A and B) Statistical significance of the data was calculated using two-way analysis of variance (ANOVA); see details in Table S1 in the supplemental material*.* (D) Quantification of *D. melanogaster* (*D*.) dead larvae and pupae, fly adults, and wasp adults on medium complemented or not, 1.5 days postinfestation, with the bacteriostatic antibiotic tetracycline. ***, *P* < 2.2 × 10^−16^; chi-square = 102.61; df = 3, using Pearson’s chi-squared test. Results are the percentages of a minimum of 270 *Drosophila* larvae. (A to D) Results are means ± SEM from three independent experiments.

*S. poulsonii* and wasp larvae coinhabit the same compartment, the hemolymph. We monitored the reciprocal impact of *S. poulsonii* on the wasp during *D. melanogaster* larval growth. We observed that wasp larvae in *S. poulsonii*-infected third-instar (L3) *D. melanogaster* larvae have the same infestation rates in the absence of *S. poulsonii* (see Fig. S1 in the supplemental material). This observation suggests that the symbiont-mediated protection takes place after the so-called “wandering larva” stage, when the larvae stop eating and initiate metamorphosis. During fly metamorphosis, the fly, wasp, and *S. poulsonii* depend upon nutrients accumulated during larval stages, since there is no food uptake at the pupal stage. Given that the *Spiroplasma* growth rate increases drastically at the pupal stage, we hypothesized that this increased growth rate negatively impacts the growth of the wasp at this critical stage (19). To test this hypothesis, we used quantitative PCR (qPCR) to monitor the growth of *L. boulardi* and *S. poulsonii* over different time periods after infestation. The growth of *L. boulardi* was completely blocked in the presence of *S. poulsonii* after the 3rd day of infestation (Fig. 2A, red line). Interestingly, the 3-day time point corresponds to *D. melanogaster* larvae entering the wandering stage. This result is consistent with a previous study indicating that *L. boulardi* wasps die during pupal stages in *Spiroplasma*-infected flies (12). Importantly, the proliferation of *S. poulsonii* was not affected by the presence of the wasp (Fig. 2B, red line). The addition of a bacteriostatic antibiotic, tetracycline, to the food of *Drosophila* larvae 2 days after infestation with *L. boulardi* (before the wandering larva stage) largely suppressed the *S. poulsonii*-mediated inhibition of wasp growth (Fig. 2C). It is noteworthy that tetracycline treatment does not eliminate *S. poulsonii* but only blocks its growth by inhibiting translation (see Fig. S2). The addition of the antibiotic markedly increased the number of emerging wasps in *S. poulsonii*-infected flies (Fig. 2D). This suggests that the capacity of *S. poulsonii* to protect against wasps depends on bacterial growth at the pupal stage. An alternative hypothesis, although less likely, is that the antibiotic inhibits the translation of a putative “anti-wasp factor.”

### *S. poulsonii* does not induce the larval cellular immune response.

The strong *S. poulsonii*-mediated protection against wasp parasites led us to examine whether this endosymbiont acts by enhancing the cellular immune response during the tripartite *S. poulsonii*-*D. melanogaster*-wasp interaction. Insects combat wasp infestation by encapsulation, a cellular immune process that involves the formation of a capsule composed of large flat hemocytes called lamellocytes. Lamellocytes stick around the developing wasp larva and are subsequently melanized by prophenoloxidase from both lamellocytes and crystal cells (33, 35, 36). Lamellocytes differentiate from 0 to 24 h postinfestation from hemocyte progenitors in the lymph gland or directly from plasmatocytes present in the circulation or in the sessile niche (37). Both melanization and hemocyte number have been shown to be involved in the level of protection (38–40).

**FIG 3:**
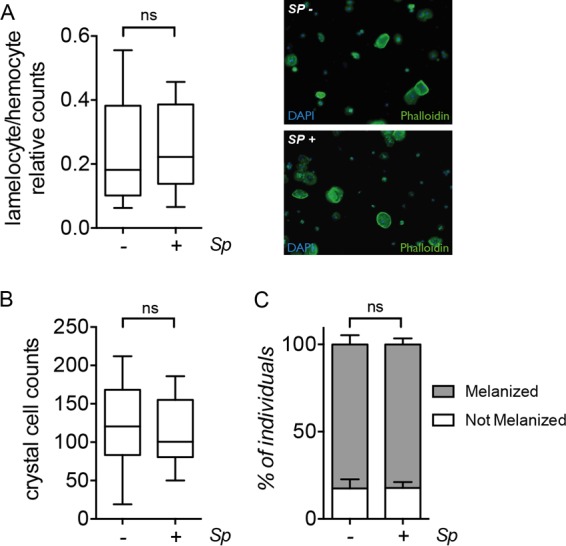
*S. poulsonii* does not affect the fly immune response. (A) Left, ratios of lamellocytes over total number of hemocytes (plasmatocytes and lamellocytes) in *D. melanogaster* flies harboring (+) or not harboring (−) *S. poulsonii* (*Sp*); right, hemocyte preparation stained with phalloidin to reveal cell shape. Nuclei of hemocytes are stained with 4[prime],6-diamidino-2-phenylindole (DAPI). Lamellocytes are identified by their large size and flat shape (white arrows). ns, *P* = 0.76; *t* = 0.3053; df = 33. (B) Crystal cell counts in whole larvae after heat treatment. ns, *P* = 0.60; *t* = 0.5260; df = 38. (C) Percentages of melanized wasp eggs or larvae. Data shown are from an experiment representative of three independent experiments. ns, *P* = 0.6110; *t* = 5363; df = 6. Statistical significance was calculated using the unpaired Student’s *t* test.

We measured the number of hemocytes in wandering larvae 72 h postinfection with *L. boulardi* and did not observe any differences from the uninfected counterpart (Fig. 3A). *S. poulsonii* also did not affect the total number of crystal cells upon infestation with *L. boulardi* (Fig. 3B). Moreover, we did not observe any difference in melanization rates or capsule formation between *D. melanogaster* larvae that were infected or not infected with *S. poulsonii* (Fig. 3C). This result indicates that *S. poulsonii* protection is not likely to be mediated by an amplification of the fly cellular immune response.

### *S. poulsonii* and *L. boulardi* consume hemolymphatic lipids.

Most parasitoid wasps, including *L. boulardi*, do not have the capacity to synthetize lipids *de novo* and rely on their host (41). We have previously shown that *Spiroplasma* growth in adult flies relies on host lipids, notably diacylglycerols (DAGs) and sterol (29). This raises the possibility that *S. poulsonii* and parasitoid wasps might compete for host lipids. In *Drosophila* larvae, the main circulating lipids are DAGs. DAGs are synthesized in the intestine from dietary lipids and then loaded with phosphoethanolamine, sterol, and other minor lipids on the apolipophorin (Lpp) vesicles. Lpp is a lipoprotein produced by the fat body (42). Lipid vesicles ensure lipid transport from the intestine to other organs. Lpp is the main hemolymph lipid carrier, since more than 95% of the hemolymph lipids in *Drosophila* cofractionate with Lpp (43). While DAGs are the predominant circulating lipids in the hemolymph, lipids are stored in the fat body as triacylglycerols (TAGs), which are produced from hemolymphatic DAGs (44).

**FIG 4:**
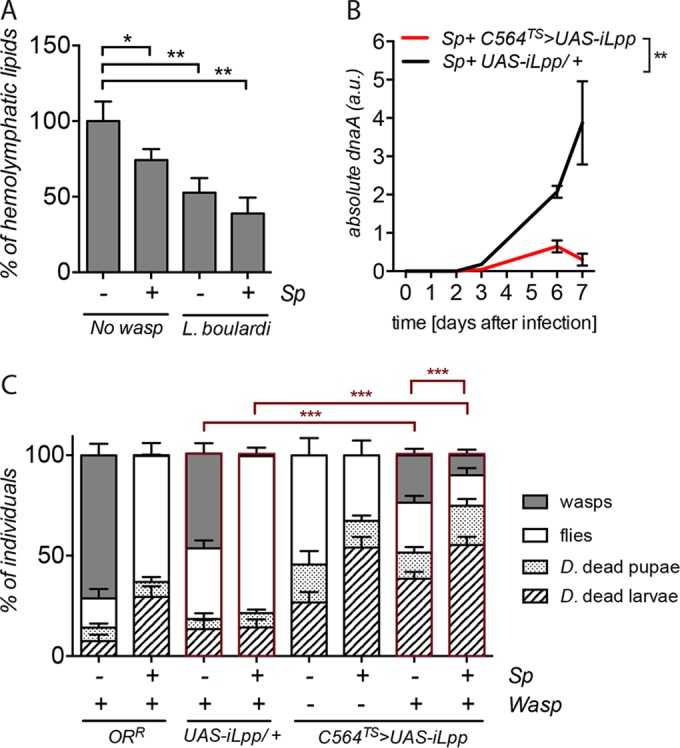
*S. poulsonii* and wasps compete for hemolymph lipids. (A) Quantification of hemolymphatic DAGs in *D. melanogaster* flies with or without wasp infestation and harboring (+) or not harboring (−) *S. poulsonii* (*Sp*). *, *P* = 0.0041; *t* = 3.268; df = 19; **, *P* = 0.0450; *t* = 2; df = 18; **, *P* = 0.0020; *t* = 3.612; df = 18; using unpaired Student’s *t* test. (B) Absolute quantification of *S. poulsonii* titers by qPCR. **, *P* = 0.00286; two-way ANOVA; see Table S1 in the supplemental material for details. (C) Fly survival after Lpp knockdown mediated by the activation of *UAS-iLpp* in the fat body using a specific thermosensitive driver (*C564-Gal4^TS^*, *C564^TS^*>). *UAS-iLpp* in the absence of driver (*UAS-iLpp/+*) and Oregon-R (*OR^R^*) flies were used as negative controls. (C) *D. melanogaster* (*D*.) dead larvae and pupae, emerging fly adults, and wasp adults. Results are percentages of a minimum of 270 *D. melanogaster* larvae. ***, from left to right, respectively: *P* < 2.2 × 10^−16^; chi-square = 153.96; df = 3; *P* < 2.2 × 10^−16^; chi-square = 620.75; df = 3; *P* < 2.2 × 10^−16^; chi-square = 84.458; df = 3; using Pearson’s chi-square test. (A to C) Results are means ± SEM from at least three independent experiments.

The impact of *S. poulsonii* on hemolymphatic lipid has never been investigated at the larval stage. We therefore monitored the amount of DAGs in hemolymph samples from third-instar larvae infected or not infected with *S. poulsonii*. The results shown in Fig. 4A indicate a reduction of DAG levels in *Spiroplasma*-infected wandering larvae compared to the levels in their uninfected counterparts. As the hemolymph extract we used to monitor the DAGs also contained *S. poulsonii*, this depletion of DAGs was detectable because DAGs are metabolized by *S. poulsonii* into cardiolipins, as previously reported in adult flies (29). We conclude that *Spiroplasma* depletes hemolymphatic DAG levels in larvae. We next investigated the levels of hemolymphatic lipids in larvae infested by *L. boulardi*. The results in Fig. 4A show that the presence of the wasp also depleted the quantity of DAGs in the hemolymph extract to a level comparable to that observed with *S. poulsonii*. It is noteworthy that when both *S. poulsonii* and the wasp were present, they had a cumulative effect on lipid depletion. In the presence of both the endosymbiont and the parasitoid, hemolymphatic lipids were decreased by about 50% compared to the amount in the control (Fig. 4A, compare 1st to 4th bar). These results, together with those of previous studies (29, 41), are consistent with the notion that both *S. poulsonii* and the wasp consume hemolymphatic lipids of *D. melanogaster* larvae to sustain their growth.

### Depletion of host lipid constrains the growth of *S. poulsonii* and *L*. *boulardi*.

We then analyzed whether reduction of host lipids affects the growth of *S. poulsonii* and *L. boulardi*. For this, we used the *Gal4^TS^/UAS* system combined with *Lpp* RNA interference (*Lpp RNAi*) to knock down Lpp in the fat body of *Drosophila* larvae (genotype, *C564-Gal4^TS^* > *UAS-iLpp*). Palm et al. have already shown that the silencing of *Lpp* in the fat body reduces the amount of circulating lipids in larvae, resulting in pupal lethality at 25°C (42). To circumvent this lethality, *Lpp RNAi* embryos were raised at 18°C during the first 4 days and then switched as L2 larvae to 25°C. As previously shown in adult flies (29), the growth of *S. poulsonii* was blocked when Lpp was depleted in larvae (Fig. 4B). Depletion of Lpp also reduced the success of *L. boulardi* after *D. melanogaster* infestation independently of the presence of *S. poulsonii*: the wasp success rate dropped from 46% in the wild type to 27% in *Lpp RNAi*-treated flies (corresponding to a 30% decrease of the fly/wasp ratio) (Fig. 4C, compare 3rd to 7th bar).

Finally, we monitored the impact of Lpp depletion on *S. poulsonii*-mediated protection against wasps. Strikingly, Lpp depletion led to a strong reduction in the *S. poulsonii*-induced protection against parasitoid wasps (Fig. 4C, 8th bar). This is probably a consequence of the impact of Lpp depletion on the growth of *S. poulsonii* (Fig. 4B). *S. poulsonii*-infected, *Lpp RNAi*-treated individuals infested with *L. boulardi* also exhibited significant lethality at the larval and pupal stages, indicating that the depletion of lipid by Lpp in the presence of *S. poulsonii* and wasps had severe negative consequences for *D. melanogaster* pupal development (Fig. 4C, compare 4th to 8th bar). These experiments demonstrate that the growth of both *S. poulsonii* and *L. boulardi* was constrained after Lpp knockdown, likely due to lipid depletion.

## DISCUSSION

Several studies recently added *Spiroplasma* to the list of inherited symbionts that confer host protection against natural enemies, with strains infecting *D. hydei* (strain SPHY) and *D. neotestacea* shown to protect against parasitoid wasps and parasitic nematodes, respectively (11, 12). These observations suggest that *Spiroplasma*-mediated protection may be common in the wild, where it could contribute to the maintenance of this symbiont in insect populations, especially under conditions of high parasite pressure (45). Our study extends some of these results by showing that *S. poulsonii* strain MSRO exerts a strong protective effect against the parasitoid wasp *L. boulardi* in two *D. melanogaster* strains (32). We further show that this protection is also observed against two distantly related wasp genera, *Leptopilina* and *Asobara*, which belong to the *Figitidae* and *Braconidae* families, respectively. *L. boulardi*, *L. heterotoma*, and *A. tabida* share the same habitats, infecting frugivorous *Drosophila* flies and, in some cases, even coinfecting the same populations (34). While *L. boulardi* is a specialist of *D. melanogaster*, *A. tabida* and *L. heterotoma* can infect up to nine *Drosophila* species. These parasitoid wasps use very distinctive infection strategies (46). Both *L. boulardi* and *L. heterotoma* have been reported to alter encapsulation by injecting venom-containing virus-like particles that target lamellocytes (33, 47). In contrast, *A. tabida* lacks virus-like particles and might be protected from encapsulation by the nature of the egg exochorion, which is sticky, allowing the egg to be embedded in host tissue (48). *Spiroplasma*-mediated protection is functional against a diversity of parasitoid species with diverse infection strategies. This leads us to favor a more generic protective mechanism.

It is noteworthy that, while *S. poulsonii* exerts some protection against three parasitoid wasp species, the outcome of the *S. poulsonii-D. melanogaster*-wasp tripartite interaction differs according to the wasp species. The presence of *S. poulsonii* markedly increases fly success upon *L. boulardi* infestation, while it leads to increased larval and pupal lethality upon *L. heterotoma* and *A. tabida* infestation. Although the former is more beneficial to the fly, high larval and pupal lethality blocks the transmission of wasps to the next generation (32). These differences are likely to be explained by differences in virulence and growth among the wasp strains.

We next investigated how *Spiroplasma* protects against parasitoid wasps and show that, consistent with a previous study, parasitoid death takes place during metamorphosis (12). Interestingly, symbiont-mediated protection correlates with symbiont growth, as protection was not observed when animals were treated with a bacteriostatic antibiotic. Quantification of a number of immunological markers relevant to encapsulation did not reveal any significant impact of *S. poulsonii* on the cellular response. This is in agreement with observations that *Spiroplasma* is not detected by the *Drosophila* immune system (19). While some studies initially suggested a role of symbionts in priming the immune system, further studies reveal that this is not likely to be the case in native host-endosymbiont interactions. Notably, *Spiroplasma* and *Wolbachia* have little, if any, effect on the immune gene expression of their native hosts (19–21).

We have recently shown that *S. poulsonii* consumes hemolymphatic lipids in adult flies, resulting in a depletion of hemolymph DAGs and a decrease of TAG storage in the fat body (29). While most lipids are directly incorporated into cell membranes in *Spiroplasma*, DAGs are transformed into cardiolipins. Metabolic analysis has suggested that parasitoid wasps are also dependent on *Drosophila* lipids (41). Some parasitoids have been reported to induce fat body lipogenesis via a specialized large cell type, the teratocyte, deriving from the extraembryonic tissue of the wasp (49). Others induce the release of lipid particles that are phagocytized by the hemocytes, and then lipid-filled hemocytes are ingested by the developing wasp larvae (50). In this work, we provide three important observations that support the notion that *S. poulsonii* bacteria and wasp larvae compete for host lipids. First, both *S. poulsonii* bacteria and *L. boulardi* wasp larvae decrease hemolymph lipids in *D. melanogaster* larvae, consistent with the notion that they consume host lipids. Second, *S. poulsonii* growth is not affected by the presence of the wasps, indicating that when *S. poulsonii* bacteria are present, the wasp larvae will develop in a lipid-depleted environment. Third, the depletion of hemolymphatic lipids using the *Lpp RNAi* construct reduces wasp success in larvae not infected with *S. poulsonii* bacteria and reduces *S. poulsonii*-mediated protection by blocking *S. poulsonii* growth in infected larvae.

Collectively, this supports a model in which lipid depletion due to the growth of *S. poulsonii* prevents efficient development of the wasp. Depletion of lipids would explain the broad-spectrum protection by *S. poulsonii* against diverse parasitoid wasps. We suspect that this protection could apply to other endosymbionts, notably *Wolbachia* bacteria. It is well known that lipid droplets interact with various organelles, including mitochondria (51). Taking into consideration the endosymbiontic resemblance between *Wolbachia* and mitochondria, *Wolbachia* might deplete lipid droplets that are required both for virus envelope formation and autophagy, an antiviral response (52). Thus, the depletion of lipid droplets by *Wolbachia* could be a potential explanation for the protection exerted by intracellular symbionts against viruses. The metabolic competition hypothesis for *Wolbachia*-mediated viral protection is supported by a study showing that *Wolbachia*-infected flies reared in cholesterol-enriched medium die faster after viral infection (25). In this study, the authors suggest that by using cholesterol as a key component of its own membrane, *Wolbachia* might deplete host cells of this lipid and, thus, interfere with viral cell entry and replication.

Consistent with our model, we observed that parasitoid wasp success is reduced when *D. melanogaster* larvae are grown on a poor diet (see Fig. S3 in the supplemental material). Attempts to improve wasp success by injecting lipids failed, either because the nature of the lipids involved is too complex or the parasitoid wasp development is too sensitive to these kinds of manipulation. Attempts to modify the *S. poulsonii*-*D. melanogaster*-wasp tripartite interaction by affecting fly nutrition also failed, likely as a consequence of *D. melanogaster*’s metabolic versatility. The dynamic nature of the *Spiroplasma*-*Drosophila*-wasp tripartite interaction over time suggests that this metabolic competition results from a complex interplay. We previously reported that *S. poulsonii* specifically depletes certain DAG species (C_16:0_ and C_18:1_) (29). We could speculate that, whereas *S. poulsonii* utilizes (and perhaps fully depletes) specific lipids, the wasp might require a broad range of lipids but not fully deplete any single class. This may explain why *S. poulsonii* affects wasp growth severely but the opposite is not the case.

Our analysis does not eliminate a role of toxin in *S. poulsonii*-mediated protection against wasps. Analysis of the *Spiroplasma* genome reveals a number of candidates, including a chitinase and five putative proteins, with low homology with the RIP contained by the *D. neotestacea Spiroplasma* that has been implicated in protection against nematodes (24, 53). Interestingly, *Spiroplasma poulsonii* MSRO did not protect *D. neotestacea* against the entomopathogenic nematode when transferred by hemolymph injection, while the *Spiroplasma* naturally found in *D. neotestacea* does protect against both parasitoid wasps and nematodes (54). These discrepancies could be explained by differences in the mechanisms of protection against distinct macroparasites. Importantly, our study does not preclude that *Spiroplasma*-mediated protection can involve, in addition to metabolic competition, the use of toxins. The contribution of each of the two mechanisms, lipid competition or toxin, could differ according to the parasite. Future research should investigate the role of such toxins and the exact nature of the lipids involved in *Spiroplasma*-wasp competition.

## MATERIALS AND METHODS

### Insect and *S. poulsonii* strains.

We used *D. melanogaster* wild-type Oregon-R fly stocks harboring or not harboring *S. poulsonii* strain MSRO but not *Wolbachia* (3, 19). We also used *D. melanogaster* Canton-S fly stocks harboring or not harboring *Spiroplasma*. The Canton-S flies both harboring and not harboring *S. poulsonii* also harbored *Wolbachia*. Canton-S fly stock harboring *S. poulsonii* was obtained by the injection of hemolymph from the infected Oregon-R flies and has been maintained in the laboratory for nearly 4 years now. To knock down the expression of *Lpp*, we used the *C564-Gal4* fat body driver in conjunction with *tubulin-Gal80^ts^* and *UAS-iLpp* (TRiP no. HM05157).

*Leptopilina boulardi* strain Lb17 was kindly provided by Michèle Crozatier. *Leptopilina Heterotoma* strain Lh14 was kindly provided by Todd Schlenke. *Asobara tabida* was kindly provided by Tadeusz J. Kawecki. All wasps were reared on Oregon-R flies at 25°C. After emergence, wasps were kept at 18°C and provided with honey. Wasps were trained for infection using L2 Oregon-R larvae before the experiments.

### Wasp infections.

*D. melanogaster* embryos were collected from 4-day-old flies by using embryo collection cages and yeasted-grape juice plates every 2 h. Once collected, embryos were maintained at 25°C for 2 days (48 h), except in experiments conducted with *C564-Gal4^ts^ UAS-iLpp* flies, and then L2 larvae were collected with a paintbrush for infection. Thirty L2 larvae were deposited on the surface of a regular corn medium vial or poor diet medium, and 4 experienced female wasps were added for 2 h. See the supplemental material in reference 30 for the composition of *Drosophila* medium. The same 4 wasps that infested the control stocks were used subsequently to infest the larvae harboring *S. poulsonii*, and vice versa.

### DNA extraction and qPCR.

We extracted DNA from 30 L2 larvae or 10 L3 larvae or pupae per sample. The DNA extraction and quantitative PCR (qPCR) protocols have been described previously (1, 55). The *Leptopilina boulardi* qPCR primers used were Lb_rRNA28qF1 (5′ GGCGAGCGAACAGGGAATA 3′) and Lb_rRNA28qR1 (5′ CCTCTATGGGTAAGTGGCCC 3′).

## SUPPLEMENTAL MATERIAL

Text S1  Supplemental materials and methods. Download Text S1, DOCX file, 0.1 MB

Figure S1  *Spiroplasma poulsonii* strain MSRO does not affect wasp infestation rates in *D. melanogaster*. (A) Rates of wasp infestation in *D. melanogaster* L3 wandering larvae harboring (*+*) or not harboring (*−*) *S. poulsonii* (*Sp*). Not significant (ns), *P* = 0.5092; *t* = 0.7471; df = 3, for comparison of L3-infested larvae. The results shown are from an experiment representative of three independent experiments. The percentages of infestation were determined by dissecting *Drosophila* larvae. Download Figure S1, PDF file, 0.1 MB

Figure S2  *S. poulsonii* growth after tetracycline treatment. Quantification of *S. poulsonii* titers relative to host DNA by qPCR during fly development. Quantification was performed as described in the legend to Fig. 2B. Statistical significance was calculated using ANOVA (significant variation among treatments) (*, *P* = 0.03509). A *post hoc* Dunnett test showed that the results with tetracycline alone differed significantly (*, *P* = 0.0498). See Table S1 for details. Download Figure S2, PDF file, 0.1 MB

Figure S3  Larvae fed on a poor diet medium are less susceptible to *L. boulardi*. Quantification of dead *D. melanogaster* larvae and pupae, fly adults, and wasp adults after *L. boulardi* infestation of *Drosophila* larvae reared on normal or poor diet medium and harboring (*+*) or not harboring (*−*) *S. poulsonii* (*Sp*). ***, *P* < 2.2 × 10^−16^; chi-square = 84.844; df = 3; using Pearson’s chi-square test. Results are percentages of a minimum of 270 *Drosophila* larvae. Download Figure S3, PDF file, 0.1 MB

Table S1  Description of the two-way ANOVA and Dunnett statistical tests performed on data from the experiments whose results are shown in Fig. 2 and Fig. 4, as well as in Fig. S2 in the supplemental material. Table S1, XLSX file, 0.04 MB
