# Supplementary material for: The Role of Lipid Competition for Endosymbiont-Mediated Protection against Parasitoid Wasps in Drosophila
Source: mBio. 2016 Jul 12;7(4):e01006-16. doi: 10.1128/mBio.01006-16 (PMC4958261; doi:10.1128/mBio.01006-16)
Supplement: Text S1 — Supplemental materials and methods. Download [file mbo004162895s1.docx]

**Supporting Information**

**Supplementary Material and Methods**

## *Lpp* knock-down and survival experiments

To knockdown the expression of *Lpp* in the larval fat body, we used the *UAS-iLpp* TRiP #HM05157 (Harvard Medical School) (42) combined with *C564-Gal4* driver in conjunction with *tubulin-Gal80^ts^*, a temperature-sensitive repressor of *Gal4* expression, which blocks *Gal4* expression at 18°C but not 25°C (56). *Spiroplasma*-infected females carrying *C564^TS^-Gal4* constructs (extended genotype, *C564-Gal4, tubP-Gal80TS*) were then crossed with males carrying *UAS-iLpp* constructs or controls (wild-type background). Over-expression of the *UAS-iLpp by C564-GAL4 at 25°C* induce lethality at the pupal stage. To circumvent this issue, *C564^TS^-Gal4/+*;*UAS-iLpp/+* embryos were maintained at 18°C until L2 instar larva and then shifted to 25°C during wasp infestation and kept at 25°C to induce (*C564^TS^-Gal4/+*;*UAS-iLpp/+)* or not *(+*;*UAS-iLpp/+)* Lpp knockdown. Lpp knockdown was confirmed by western blot (data not shown). All flies were maintained at 25°C unless otherwise specified. See Herren et al., 2014 supplemental material (30) for the composition of *Drosophila* media. For survival experiments, counts were made every 24 hours. Tetracycline was added 1.5 days after infestation, before wandering larva stage at [0.01mg/µl] final concentration on the fly medium.

## Metabolite analyses

*Drosophila* larval hemolymph was collected from 10 larvae. Larval cuticles were pierce and placed in 150µL of cold PBS. Tubes were centrifuged for 10 minutes at 4°C, 5’000 r.p.m and 100µl of supernatant was collected for subsequent analyses. Protein concentration was determined using Pierce^TM^ BCA Protein Assay Kit (Thermo Scientific). Hemolymph DAGs were analyzed using a coupled colorimetric assay (57).

## Imaging

To observe *Drosophila larval* hemocytes, larvae were bled on microscope slides in 5μl PBS containing 0.01 µg of Phenylthiourea (Invitrogen) to avoid hemolymph coagulation. Slides were incubated at 25°C for 30 min to let the cells settle at the bottom of the PBS drop, then fixed for 15 min in PBS containing 0.1% Tween 20 and 4% paraformaldehyde, then rinsed 3 times with PBS containing 0.1% Tween 20. Actin was stained using 488-conjugated phalloidin (Molecular Probes) (1: 200) and nucleus with DAPI (1:10000). Slides were then mounted and observed on an Axioimager Z1 (Zeiss). Images were captured with an Axiocam MRn camera and Axiovision software. For sessile crystal cells counts larvae were heated in PBS at 67 °C for 20 min to induce the heat activation of PPO within hemocytes (39).

## Data treatment and Statistical analysis

Statistical significance was calculated using an unpaired Students *t* test, two-way ANOVA and Dunnet, and Chi-squared (with GraphPad Prism 5.0 and R) and considered significant if *P*-values were lower than 0.05. Asterisks indicate the level of significance: * *P*<0.05, ** *P<*0.01 and *** *P*<0.001 and ns (non-significant). For growth of *Spiroplasma* and wasp tissue by *qPCR* values were log2 transformed prior to the two-way ANOVA test.
