# Supplementary figures and images for: The Role of Lipid Competition for Endosymbiont-Mediated Protection against Parasitoid Wasps in Drosophila
Source: mBio. 2016 Jul 12;7(4):e01006-16. doi: 10.1128/mBio.01006-16 (PMC4958261; doi:10.1128/mBio.01006-16)

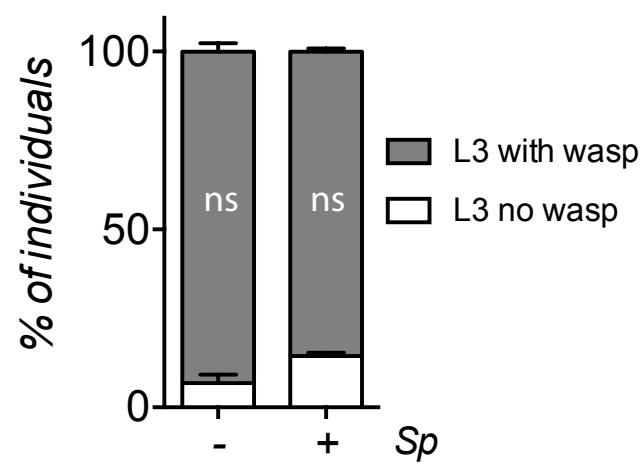

Figure S1

Supplement: Figure S1 — Spiroplasma poulsonii strain MSRO does not affect wasp infestation rates in D. melanogaster. (A) Rates of wasp infestation in D. melanogaster L3 wandering larvae harboring (+) or not harboring (−) S. poulsonii (Sp). Not significant (ns), P = 0.5092; t = 0.7471; df = 3, for comparison of L3-infested larvae. The results shown are from an experiment representative of three independent experiments. The percentages of infestation were determined by dissecting Drosophila larvae. Download [file mbo004162895sf1.pdf]

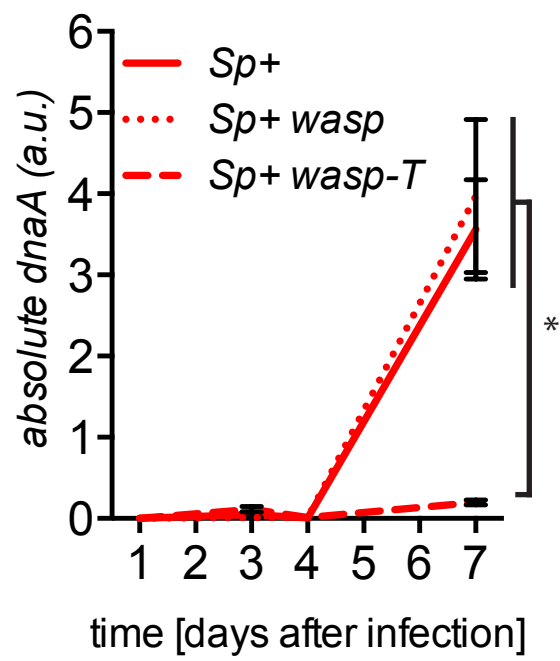

Figure S2

Supplement: Figure S2 — S. poulsonii growth after tetracycline treatment. Quantification of S. poulsonii titers relative to host DNA by qPCR during fly development. Quantification was performed as described in the legend to Fig. 2B. Statistical significance was calculated using ANOVA (significant variation among treatments) (*, P = 0.03509). A post hoc Dunnett test showed that the results with tetracycline alone differed significantly (*, P = 0.0498). See Table S1 for details. Download [file mbo004162895sf2.pdf]

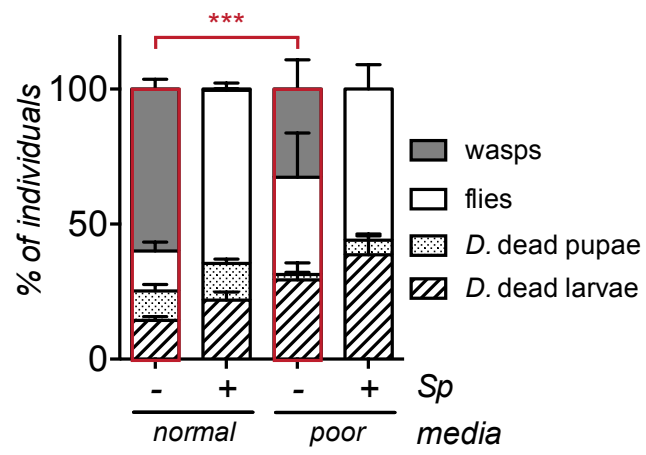

Figure S3

Supplement: Figure S3 — Larvae fed on a poor diet medium are less susceptible to L. boulardi. Quantification of dead D. melanogaster larvae and pupae, fly adults, and wasp adults after L. boulardi infestation of Drosophila larvae reared on normal or poor diet medium and harboring (+) or not harboring (−) S. poulsonii (Sp). ***, P < 2.2 × 10^−16^; chi-square = 84.844; df = 3; using Pearson’s chi-square test. Results are percentages of a minimum of 270 Drosophila larvae. Download [file mbo004162895sf3.pdf]
